# Multi‐Omics Analyses Reveal Relationships Between Gut Microbiota and Frailty

**DOI:** 10.1002/brb3.70657

**Published:** 2025-07-07

**Authors:** Xinlei Hou, Luwen Zhu, Jiongliang Zhang, Xinyue Li, Donghui Yu, Yuting Wang, Yumeng Su, Xiangyu Wei, Hanwen Ma, Wenjing Song, Jinting Li, Lili Teng, Qiang Tang, Minmin Wu

**Affiliations:** ^1^ Department of Acupuncture The Second Affiliated Hospital of Heilongjiang University of Chinese Medicine Harbin China; ^2^ Rehabilitation Center The Second Affiliated Hospital of Heilongjiang University of Chinese Medicine Harbin China; ^3^ Heilongjiang Provincial Key Laboratory of Brain Function and Neurorehabilitation Harbin China; ^4^ Department of Rehabilitation Medicine Heilongjiang University of Chinese Medicine Harbin China

**Keywords:** frailty, gut microbiota, Mendelian randomization, *TET2*

## Abstract

**Introduction:**

Observational studies suggest a strong association between gut microbiota and frailty, but the underlying mechanisms remain unclear. This study aimed to investigate potential causal links and biological pathways linking gut microbiota and frailty.

**Methods:**

We utilized summary‐level data of gut microbiota and frailty data from MiBioGen and a genome‐wide association meta‐analysis. A bidirectional, two‐sample Mendelian randomization (MR) analysis was performed to investigate the causal relationship between gut microbiota and frailty. Additional genetic and genomic analyses were conducted to identify common biological pathways.

**Results:**

We identified eight causal relationships between the gut microbiota composition and frailty. Inverse‐variance weighting suggested that genetic liability for the class Betaproteobacteria and genera *Allisonella*, *Bifidobacterium*, *Clostridium innocuum*, and *Eubacterium coprostanoligenes* was associated with increased frailty risk. In contrast, the class Bacteroidia, genus *Eubacterium ruminantium*, and the order Bacteroidales were associated with decreased risk. Reverse MR analysis provided no evidence for a causal effect of frailty on gut microbiota composition. In addition, *TET2* was identified as a key hub gene associated with frailty, potentially linking gut microbiota to immune dysregulation and aging‐related inflammatory pathways.

**Conclusions:**

Our findings provide genetic evidence that gut microbiota composition influences frailty risk and highlight *TET2* as a potential mechanistic link via immune dysregulation. These results suggest that microbiota‐targeted interventions may offer novel strategies for the prevention and management of frailty in older adults.

## Graphical abstract

This study applied bidirectional Mendelian randomization and transcriptomic analysis to explore the causal link between gut microbiota and frailty. Results revealed a unidirectional effect of microbiota on frailty and identified TET2 as a key gene potentially mediating this association through immune‐related pathways.

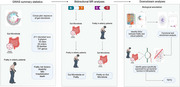

## 1 . Introduction

Global public health challenges are increasingly shaped by aging populations (Suzman et al. 2015). In 2019, approximately 9% of the global population was aged ≥65 years, a figure projected to rise to approximately 12% by 2030 and 23% by 2100 (Almohaisen et al. 2022). As the number of older adults grows, the prevalence of chronic illness and frailty increases. Frailty affects an estimated 10.7% of older adults in the community, 47.4% in hospitals, and 52.3% in nursing homes. This condition significantly reduces the quality of life and imposes a significant economic burden (Doody et al. 2022). Frailty is a reversible, multidimensional syndrome characterized by increased vulnerability to stressors due to age‐related physiological decline. It increases the risk of adverse outcomes like disability and mortality, highlighting the need for better diagnosis and care, especially through promising gut flora modifications (Poursalehi et al. 2023; Gonçalves et al. 2022; Hoogendijk et al. 2019).

The human gastrointestinal tract hosts a diverse and dynamic community of microorganisms, including bacteria, archaea, viruses, and fungi that constitute the gut microbiota. These microorganisms engage in complex and mutually beneficial interactions with the host (Huang et al. 2022). Recent genome‐wide association studies (GWAS) have found significant host–microbiome interactions between human genes and gut microbiomes (Lopera‐Maya et al. 2022; Kurilshikov et al. 2021). Although individual microbiome profiles vary, they tend to stabilize over time in healthy adults. Healthy non‐dysbiotic microbiota live in symbiosis with their hosts and promote health by providing vital protection (pathogen removal, antimicrobial production, and competition for nutrients) and improving structural (strengthening the flora barrier, neuroimmune system development) and metabolic functions (production of biotin and folate, regulation of intestinal epithelial cell differentiation and proliferation, energy preservation, carbohydrate fermentation, and ion adsorption) (Hold and Hansen 2019). However, the composition and function of microbiomes change with increasing age, adversely affecting digestion, absorption, and immune function, leading to poor health and frailty in older adults.

A recent systematic review comparing frail and healthy older adults reported that frailty is associated with reduced gut microbiota diversity and a lower abundance of short‐chain fatty acid (SCFA) producers. These changes may lead to increased gut permeability, upregulation of pro‐inflammatory cytokines, and heightened frailty risk (Rashidah et al. 2022). These observations suggest a potentially causal relationship between gut microbiota composition and frailty.

Mendelian randomization (MR) has emerged as a powerful method for inferring causality from observational data, especially with the growing availability of genetic information related to both microbiota and complex traits. According to the Mendelian laws of heredity, MR offers a distinct advantage in examining the potential causal relationship between two features (VanderWeele et al. 2014). In parallel, advances in bioinformatics have enabled a more thorough understanding of disease pathobiology at the genetic level (Lin et al. 2023).

This study aimed to investigate the causal relationship between gut microbiota composition and frailty risk using MR analysis. In addition, we explored the underlying genetic mechanisms, including key hub genes that may mediate this relationship. Our findings may help develop new treatment methods targeting the gut microbiota and prevent or mitigate frailty in older adults.

## 2 Methods

### 2.1 Study Design

**FIGURE 1:**
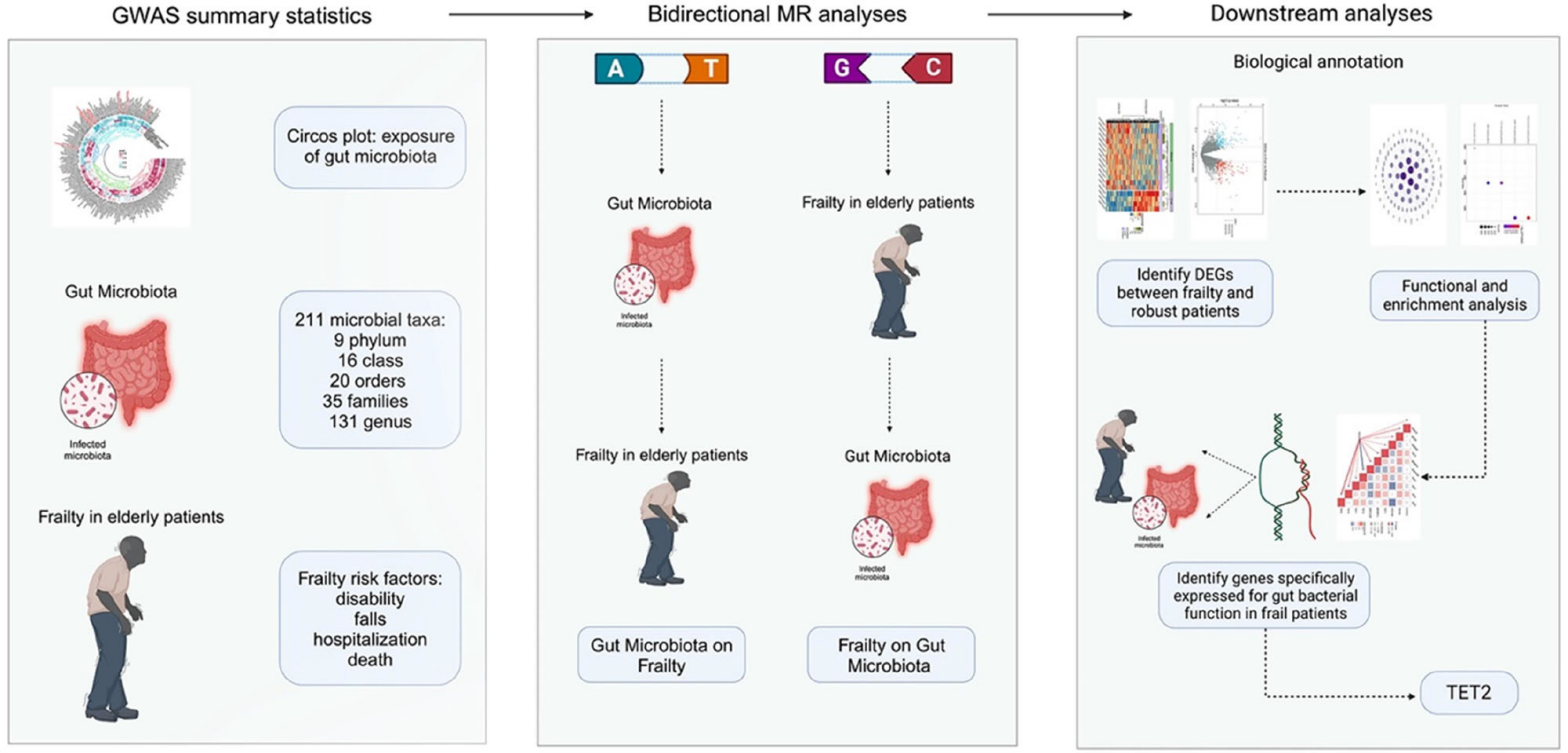
Study overview.

An overview of the methodology used in this study is presented in Figures 1. To examine the causal relationship between gut microbiota and frailty, we conducted a two‐sample MR study. The following three key assumptions must be met for a valid instrumental variable (IV) in MR: (i) Genetic variation as an IV must be significantly associated with the gut microbiome (as exposure); (ii) genetic variation must be independent of any conventional and unknown confounders; and (iii) genetic variation must be associated with frailty only through the gut microbiome (VanderWeele et al. 2014). An independent GWAS was the primary foundation for the summary data. Single nucleotide polymorphisms (SNPs) are used in MR to evaluate the causal relationship between exposure and outcome. We also performed downstream analyses by biological annotation. The study adhered to the MR‐STROBE guidelines for reporting (Skrivankova et al. 2021).

### 2.2 GWAS Data Sources

We utilized GWAS data from the MiBioGen study (https://mibiogen.gcc.rug.nl/), which analyzed 211 gut microbiota taxa across five taxonomic levels (phylum, class, order, family, and genus) in 18,340 participants from 24 cohorts (Kurilshikov et al. 2021). Microbial composition was characterized in three cohorts by targeting the V4, V3–V4, and V1–V2 regions of the 16S rRNA gene, with 10,000 readings selected per cohort. The study identified genetic variations that altered the relative abundance of microbial taxa using microbiota quantitative trait loci mapping (Kurilshikov et al. 2021).

For frailty, we utilized the largest known meta‐analysis of GWASs on this condition, combining data from the UK Biobank (UKB, *N* = 164,610) and TwinGene (*N* = 10,616), totaling 175,226 European‐ancestry participants (Atkins et al. 2021). Frailty was assessed using the Frailty Index (FI), based on the deficit accumulation model (Atkins et al. 2021; Searle et al. 2008). The UKB FI was constructed from 49 baseline self‐reported variables related to symptoms, disabilities, and diagnosed diseases, while TwinGene used 44 similar variables. Both indices were quantile‐normalized before analysis. At a genome‐wide significance threshold (*p* < 5 × 10^−8^), 14 leading SNPs at different loci associated with FI were identified as genetic instruments.

All original studies received appropriate ethical approvals, and informed consent was obtained as the data were publicly available.

### 2.3 SNP Selection

To ensure the validity and accuracy of the causal inference between gut microbiota and frailty, we applied several quality control techniques to select the proper genetic IV: First, we evaluated the relationship between IV and gut microbiota, which has a minimal number of loci, using a threshold of *p* < 1.0 × 10^−5^, consistent with that in most MR studies on gut microbiota (Kurilshikov et al. 2021; N. Li, Wang, et al. 2023). Second, to avoid the effect of linkage disequilibrium, we clumped the SNPs (*R* ^2 ^< 0.001, process distance = 10,000 kb). Third, palindromic SNPs (those with A/T or G/C alleles) were removed to prevent strand orientation or allele‐coding distortion. Fourth, to check for potential confounders, PhenoScanner (http://www.phenoscanner.medschl.cam.ac.uk/) was used to search for SNPs with positive results (Kamat et al. 2019). Finally, the instrument strength related to exposure attributes was estimated using the *F*‐statistic. If *F* < 10 was used in the MR analyses, no weak IV bias was observed owing to the lack of a significant correlation between IV and the gut microbiome.

### 2.4 MR Analysis

To identify the association of gut microbiota with frailty, MR analyses were performed using multiple methods: inverse variance‐weighted (IVW), MR‐Egger regression (sensitivity analysis), simple mode (sensitivity analysis), weighted median (sensitivity analysis), weighted mode (sensitivity analysis), and maximum likelihood. We used IVW as the main technique, assuming all SNPs were accessible as reliable variables (Bowden et al. 2016). The potential pleiotropy of the SNP was estimated using the MR‐Egger intercept, with a *p* value > 0.05 suggesting no horizontal pleiotropy (Bowden et al. 2015; Verbanck et al. 2018). We also calculated Cochrane *Q* values to evaluate heterogeneity. The leave‐one‐out sensitivity method was employed to ascertain whether a particular genetic locus affected random estimates. Scatter and forest plots were constructed to further highlight the sensitivity of the findings.

We then performed a reverse MR analysis using frailty‐associated SNPs as IVs, with significant bacterial as outcomes and frailty as the exposure, to examine whether frailty had any causal effect. To determine whether the exposure had a directional causal effect on the outcome, we used the MR Steiger directionality test (Hemani et al. 2017).

All calculations were performed using the “TwoSampleMR” package in R (version 4.2.2), with findings reported as odds ratios and 95% confidence intervals. A *p* value < 0.05 was considered statistically significant (Hemani et al. 2018; Yavorska and Burgess 2017).

### 2.5 Biological Annotation

#### 2.5.1 Frailty Microarray Data and Gut Microbiota‐Regulated Genes

Human frailty‐related gene expression data was searched in NCBI's Gene Expression Omnibus database (https://www.ncbi.nlm.nih.gov/geo/) using “frailty” and “Homo sapiens” as keywords. The eligible dataset was GSE140358, comprising normalized log₂‐transformed expression matrix data from peripheral blood samples of 25 frail and 25 robust individuals. To further correct for batch effects and unwanted variation, surrogate variable analysis (SVA) was applied using the “sva” package in R. GSE140358 blood samples were obtained from frail and robust patients. Differentially expressed genes (DEGs) were screened using the “limma” package (V3.44.3) of the R software program (version 4.2.2) with the thresholds |logFC| >1 and *p* < 0.05. Volcano plots of DEGs were generated using the “ggplot2” package (V3.3.2).

Furthermore, we searched the MSigDB database (https://www.gsea‐msigdb.org/gsea/index.jsp) to identify microbe‐related markers, drivers, and other functional genes (keywords: “bacteria” or “gut microbiota”).

#### 2.5.2 Functional Enrichment Analysis and Protein–Protein Interaction (PPI) Network Construction

We conducted functional enrichment analysis of DEGs using the Database for Annotation, Visualization, and Integrated Discovery (DAVID; https://david.ncifcrf.gov/). The DEG list, annotated with “Official Gene Symbols,” was uploaded into DAVID with the species limited to “Homo sapiens.” Gene Ontology (GO) terms, including biological process (BP), cellular component (CC), and molecular function (MF), as well as Kyoto Encyclopedia of Genes and Genomes (KEGG) pathways, were analyzed. A *p* value < 0.05 was considered statistically significant. Subsequently, SangerBox (version 1.1.3; http://vip.sangerbox.com/) was employed to visualize the top five significantly enriched GO terms and KEGG pathways based on gene count using bubble charts.

A PPI network was constructed by importing DEGs into the Search Tool for the retrieval of Interacting Genes/Proteins database (http://string‐db.org), with a PPI score >0.4. The resultant network data was visualized in Cytoscape software (version 3.7.2). In the network, each node represents a protein, and each edge represents an interaction between two proteins. A grid layout algorithm was applied in Cytoscape to clearly organize the nodes and improve network readability. Hub genes were subsequently identified using the CytoHubba plug‐in of Cytoscape. Nodes were ranked according to their maximal clique centrality (MCC) scores, which reflect the extent of a node's participation in maximal cliques within the network (Chin et al. 2014). The MCC algorithm was applied with default settings, and the top‐ranked genes were selected as hub genes for further analysis.

#### 2.5.3 Intestinal Microbiome‐Related Functions and the Correlation with Hub Genes

To reveal the gut microbiota profiles of frail patients, microbial gene scores were calculated from MsigDB using the single‐sample Gene Set Enrichment Analysis (ssGSEA) function of the R package GSVA. Spearman's correlation analysis was used to assess the correlation between gut microbiota scores and hub genes.

## 3 Results

### 3.1 Genetic Instruments for Gut Microbiome

We identified 211 bacterial features across five taxonomic levels: phylum, class, order, family, and genus. For each bacterial feature, comprehensive information on the resulting SNPs (including effect allele, other allele, beta, standard error, *p* value, and *F*‐value) is provided in Table S1.

### 3.2 Causal Effects of the Gut Microbiome on Frailty

**FIGURE 2:**
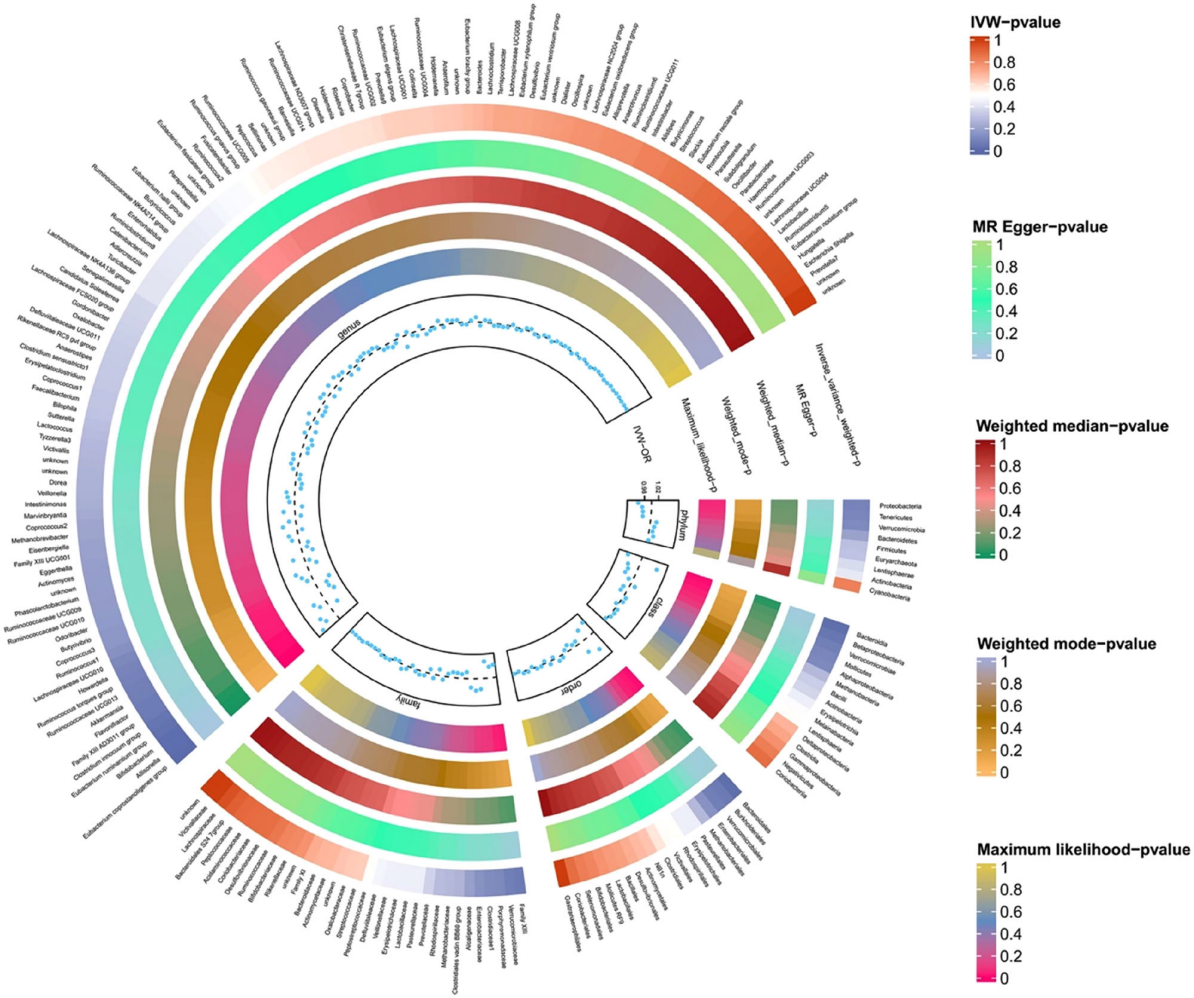
Circos plot for gut microbiota and frailty. The outer layer of the Circos plot shows the exposure of all gut microbiota and the inner layer of the Circos plot shows five layers, each representing one MR method. The colors represent *p* values.

**FIGURE 3:**
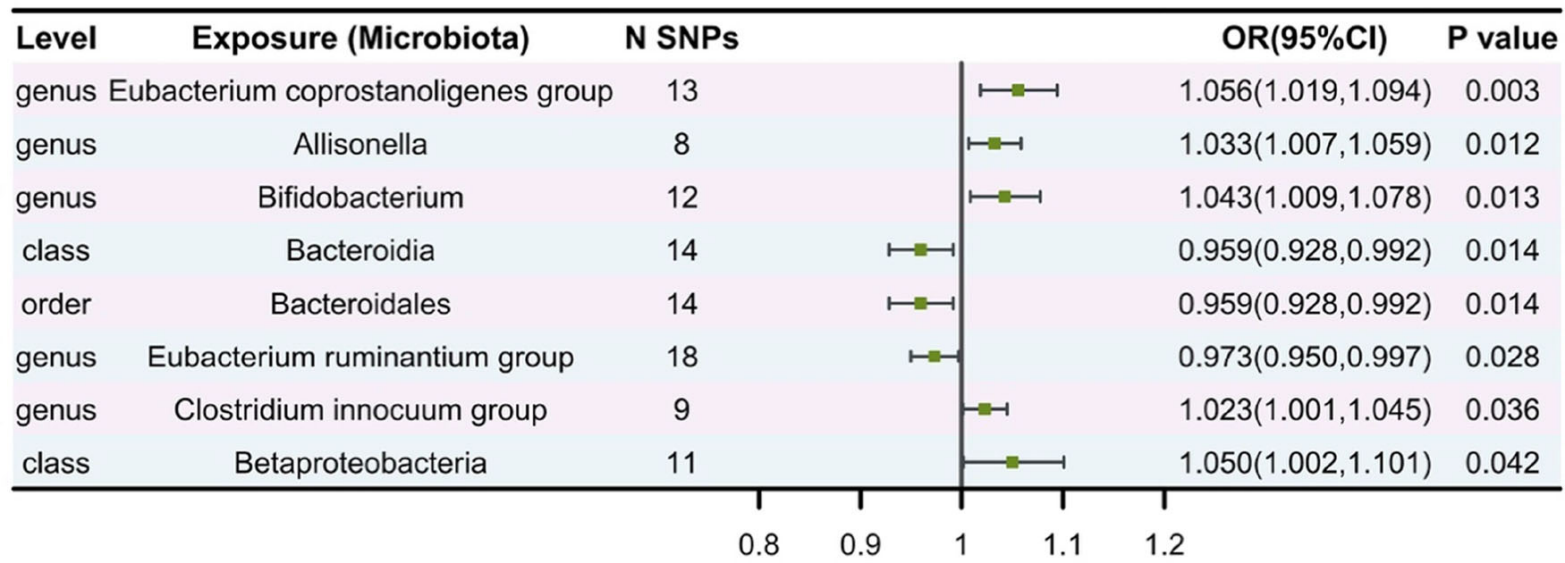
MR analysis. Causal effects were estimated using primary analysis IVW. The forest plot displays potential causal relationships between gut microbiota and frailty. Data are presented as odds ratios (OR), with corresponding 95% confidence intervals (CI).

**FIGURE 4:**
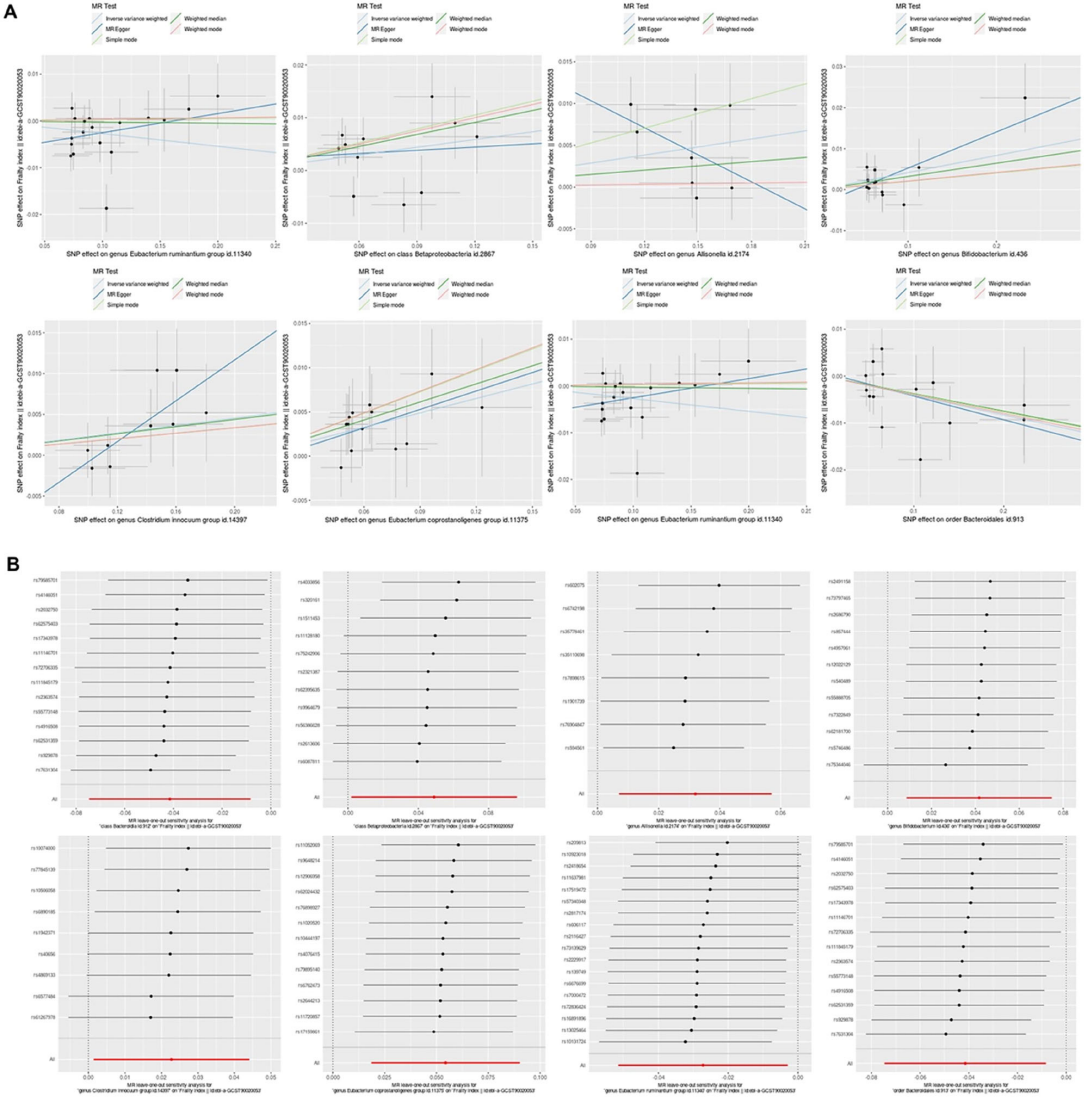
**(A)** MR scatter plots. Scatterplot of the potential effects of single‐nucleotide polymorphisms (SNPs) on gut microbiota (class Betaproteobacteria, genus *Allisonella*, genus *Bifidobacterium*, genus *Clostridium innocuum* group, genus *Eubacterium coprostanoligenes* group, class Bacteroidia, genus *Eubacterium ruminantium* group, and order Bacteroidales) versus frailty. The slope of each line represents the causal effect estimate using the corresponding MR analysis model, and the intercept can be interpreted as an estimate of the average horizontal pleiotropic effect across the genetic variants. **(B)** Leave‐one‐out plots for MR results. Leave‐one‐out plots for the causal effects of gut microbiota on frailty.

IVW analyses revealed that class Betaproteobacteria (OR = 1.05, 95% CI, 1.00–1.10, *p* = 4.2e‐02), genera *Allisonella* (OR = 1.03, 95% CI, 1.01–1.06, *p* = 1.2e‐02), *Bifidobacterium* (OR = 1.04, 95% CI, 1.01–1.08, *p* = 1.3e‐02), *Clostridium innocuum* (OR = 1.02, 95% CI, 1.00–1.05, *p* = 3.6e‐02), and *Eubacterium coprostanoligenes* (OR = 1.06, 95% CI, 1.02–1.09, *p* = 2.9e‐03) groups were potentially associated with an increased risk of frailty. In contrast, the class Bacteroidia (OR = 0.96, 95% CI, 0.93–0.99, *p* = 1.4e‐02), genus *Eubacterium ruminantium* (OR = 0.97, 95% CI, 0.95–1.00, *p* = 2.8e‐02), and order Bacteroidales (OR = 0.96, 95% CI, 0.93–0.99, *p* = 1.4e‐02) were associated with a lower risk of frailty (Table 1; Figures 2 and 3). Detailed effect estimates from all MR methods are provided in Table S2. As shown in Table 1, the sensitivity analyses did not show clear evidence of potential horizontal pleiotropy (all *P*_for Egger intercept_ > 0.05). Heterogeneity was not observed among the SNPs (all *P*_for Cochran's Q_ > 0.05). Figure 4 shows additional visualization outcomes, including scatterplots and leave‐one‐out plots.

**TABLE 1:** Significant Mendelian randomization (MR) results of the causal relationships between gut microbiome and frailty based on inverse‐variance weighted (IVW) method.

Exposure	No. SNP	Methods	*β*	SE	*P*_IVW_	OR (95% CI)	Horizontal pleiotropy *P*_for Egger intercept_	Heterogeneity *P*_for Cochran’s Q_	Correct causal direction	Steiger *p* value
Class Bacteroidia	14	IVW	−0.041	0.017	1.4e‐02	0.96 (0.93–0.99)	0.778	0.368	True	0.499
Class Betaproteobacteria	11	IVW	0.049	0.024	4.2e‐02	1.05 (1.00–1.10)	0.738	0.131	True	0.512
Genus *Allisonella*	8	IVW	0.032	0.013	1.2e‐02	1.03 (1.01–1.06)	0.098	0.111	True	0.592
Genus *Bifidobacterium*	12	IVW	0.042	0.017	1.3e‐02	1.04 (1.01–1.08)	0.285	0.795	True	0.500
Genus *Clostridium innocuum* group	9	IVW	0.023	0.011	3.6e‐02	1.02 (1.00–1.05)	0.102	0.567	True	0.581
Genus *Eubacterium coprostanoligenes* group	13	IVW	0.054	0.018	2.9e‐03	1.06 (1.02–1.09)	0.835	0.979	True	0.500
Genus *Eubacterium ruminantium* group	18	IVW	−0.027	0.012	2.8e‐02	0.97 (0.95–1.00)	0.116	0.102	True	0.457
Order Bacteroidales	14	IVW	−0.041	0.017	1.4e‐02	0.96 (0.93–0.99)	0.778	0.368	True	0.499

Abbreviations: CI, confidence intervals; No. SNP, number of single‐nucleotide polymorphism; OR, odds ratio; SE, standard error.

**FIGURE 5:**
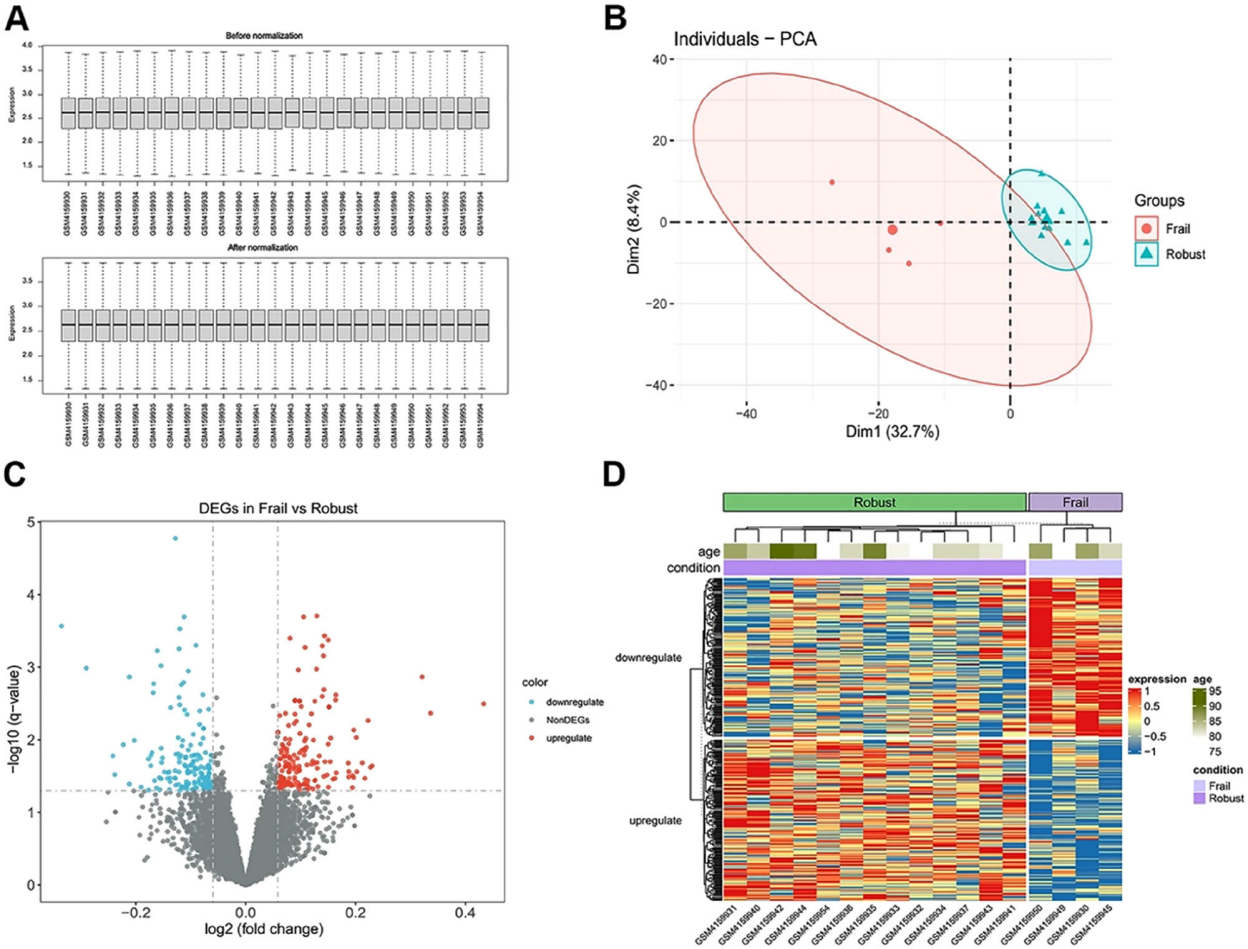
Post‐correction boxplots and principal component analysis (PCA) plots showing consistent biology‐driven clustering. **(A)** Post‐correction boxplots. **(B)** PCA results. **(C, D)** Volcano plot and heatmap showing differentially expressed genes (DEGs) using limma analysis.

**FIGURE 6:**
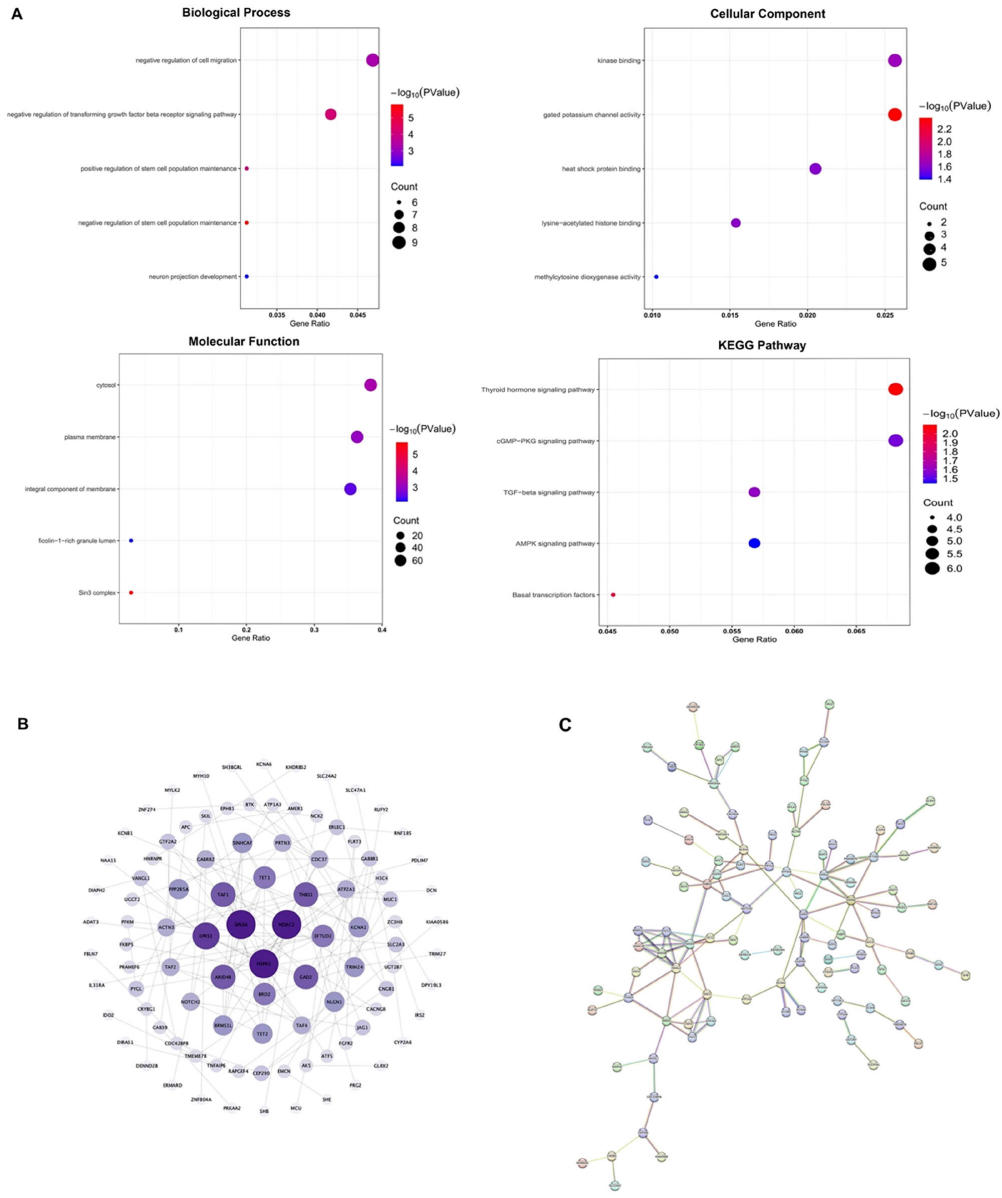
Functional analysis of DEGs. **(A)** Results of Gene Ontology (GO) and Kyoto Encyclopedia of Genes and Genomes (KEGG) enrichment analyses. **(B)** In the protein–protein interaction network, the darker the color, the greater the number of neighboring nodes. **(C)** PPI network with 97 nodes and 133 edges. Edge colors in the STRING database's predicted PPIs indicate multiple kinds of evidence supporting the prediction.

**FIGURE 7:**
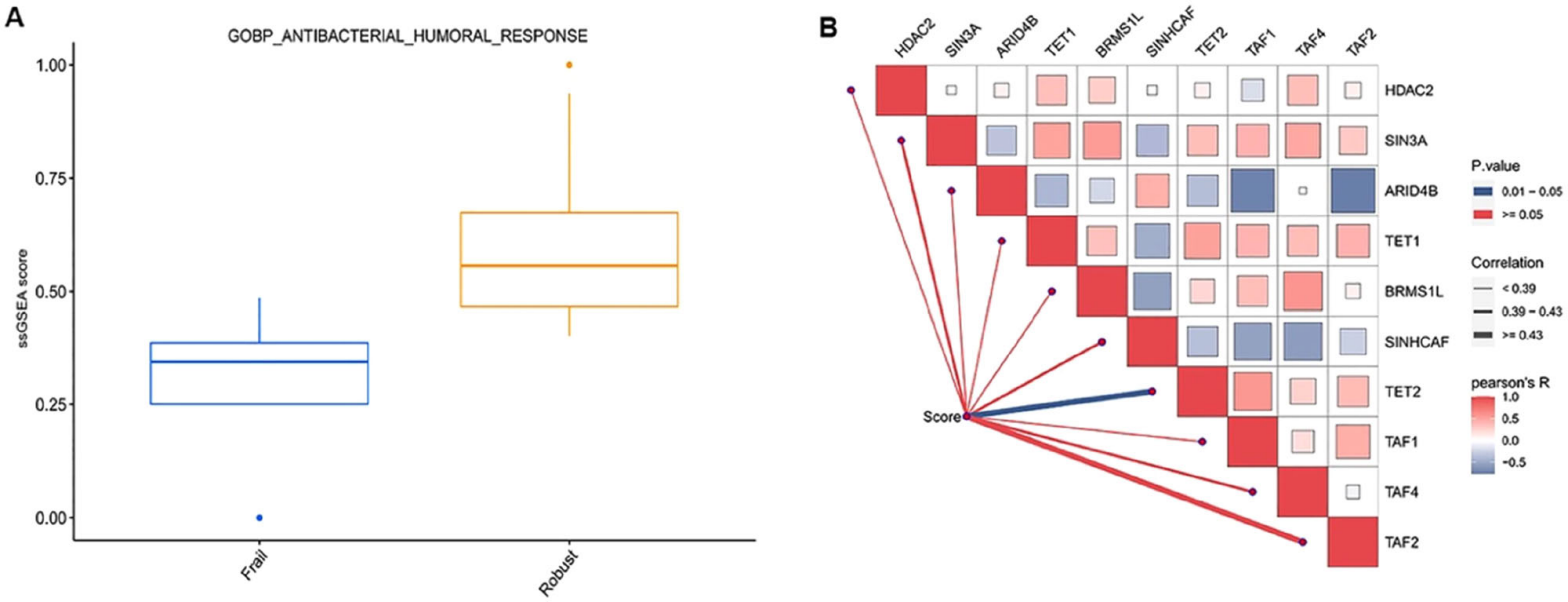
Development of gut flora‐related scores in frail older patients. **(A)** The difference in the antibacterial function between frailty and robust samples. **(B)** The relationships between gut microbe‐related function scores and hub genes in frail older patients.

### 3.3 Causal Effects of Frailty on the Gut Microbiome

To assess reverse causality, we used frailty as the exposure and gut microbiota as the outcome. In the reverse MR analysis, nine genome‐wide significance levels (*p* < 5 × 10^−8^) and independent SNPs were proposed as IVs for frailty (Table S3). We found that genetically predicted frailty did not significantly affect the development of class Betaproteobacteria, genus *Allisonella*, *Bifidobacterium*, *C. innocuum*, *E. coprostanoligenes*, class Bacteroidia, *E. ruminantium*, or order Bacteroidales, in the absence of heterogeneity and pleiotropy (except for Betaproteobacteria, heterogeneity *p* for Cochran's *Q* = 0.024 < 0.05) (Table 2).

**TABLE 2:** MR results of causal relationships between frailty and the gut microbiota.

Exposure	Outcome	No. SNP	Methods	*β*	SE	*P*_IVW_	OR (95% CI)	Horizontal pleiotropy *P*_for Egger intercept_	Heterogeneity *P*_for Cochran’s Q_	Steiger *p* value	Correct causal direction
Frailty	Class Bacteroidia	7	IVW	−0.010	0.178	0.954	0.99 (0.70–1.40)	0.8	0.783	0.93	True
	Class Betaproteobacteria	7	IVW	0.364	0.263	0.166	1.44 (0.86–2.41)	0.866	0.057	0.988	True
	Genus *Allisonella*	6	IVW	−0.113	0.448	0.801	0.89 (0.37–2.15)	0.717	0.618	0.938	True
	Genus *Bifidobacterium*	7	IVW	0.053	0.199	0.789	1.05 (0.71–1.56)	0.314	0.968	0.928	True
	Genus *Clostridium innocuum* group	7	IVW	0.279	0.374	0.455	1.32 (0.64–2.75)	0.399	0.771	0.935	True
	Genus *Eubacterium coprostanoligenes* group	7	IVW	−0.056	0.216	0.794	0.95 (0.62–1.44)	0.126	0.214	0.952	True
	Genu *Eubacterium ruminantium* group	7	IVW	−0.010	0.276	0.972	0.99 (0.58–1.70)	0.61	0.557	0.936	True
	Order Bacteroidales	7	IVW	−0.010	0.178	0.954	0.99 (0.70–1.40)	0.8	0.783	0.93	True

Abbreviations: CI, confidence intervals; No. SNP, number of single‐nucleotide polymorphism; OR, odds ratio; SE, standard error.

### 3.4 Post‐Correction Boxplots and Principal Component Analysis Plots

After correction, the boxplots showed more homogeneous and comparable distributions of gene expression values across the sample groups (Figures 5A). Principal component analysis results showed the close clustering of the two sample groups (Figures 5B). Differential expression analysis revealed 171 significantly upregulated and 168 downregulated transcripts in the blood of frail patients compared to robust controls (Figures 5C,D). The complete list of DEGs is provided in Table S4.

### 3.5 GO and KEGG Enrichment Analysis

Using the DAVID tool, we found that 60 GO and 5 KEGG terms were significantly enriched in the DEGs (*p* < 0.05). The top five enriched GO terms were categorized into three categories: BP, MF, and CC. Our findings also revealed the top five KEGG pathway terms (Figures 6). The BP terms were related to the regulation of cell migration, transforming growth factor beta receptor signaling pathway, stem cell population maintenance, and neuron projection development. In the CC annotation, DEGs were mainly involved in the cytosol, plasma membrane, membrane integral components, ficolin‐1‐rich granule lumen, and the Sin3 complex. The most enriched MF terms were kinase‐binding‐gated potassium channel activity, heat shock protein binding, lysine‐acetylated histone binding, and methylcytosine dioxygenase activity. The most significant pathway was the thyroid hormone signaling pathway (*p* = 0.008). The remaining highly enriched pathways were basal transcription factors, transforming growth factor beta signaling pathway, cGMP‐PKG signaling pathway, and AMPK signaling pathway. Detailed enrichment results are listed in Table S5.

### 3.6 PPI and Hub Genes and Their Correlation with Gut Bacteria Functions

We mapped the DEGs to PPIs and identified 97 nodes and 133 edges. In addition, 10 hub genes were identified using the MMC plugin in Cytoscape, including *HDAC2*, *SIN3A*, *ARID4B*, *TET1*, *BRMS1L*, *SINHCAF*, *TET2*, *TAF1*, *TAF4*, and *TAF2* (Table S6). The ssGSEA analysis showed significant antimicrobial function in frail versus robust patients (Figures 7A). Spearman correlation analysis showed a significant correlation between the gut microbiome‐related function scores and *TET2* gene expression (Figures 7B).

## 4 Discussion

This MR study showed that specific gut microbiota genetic profiles are linked to frailty development, with the analysis minimizing confounding factors like lifestyle and socioeconomic status using genetic markers (Davies et al. 2018). We also examined the biological relationship between gut microbes and frailty through multi‐omics data analysis. Gut dysbiosis is characterized by reduced microbiota diversity and the rise of harmful pathogens, which are generally harmful. In contrast, higher diversity is linked to better health and reduced inflammatory diseases (Sommer et al. 2017; Lim et al. 2017; Zuo et al. 2018). In community‐dwelling older adults, lower gut microbiota diversity has been correlated with frailty, although it remains unclear if frailty causes or results from reduced diversity, and the specific impactful taxa are yet to be identified (Rashidah et al. 2022; Almeida et al. 2022).

Our findings align with previous studies indicating a potential role for Betaproteobacteria in frailty. Studies on the intestinal flora of aging humans and mice have revealed a significant increase in the abundance of Proteobacteria (Leite et al. 2021), which may be associated with the release of pro‐inflammatory factors, a loss of microbial diversity (Bárcena et al. 2019), and a decline in the regulatory functions of the metabolic and immune systems (Adriansjach et al. 2020). Furthermore, a study on the effects of aging on the fecal microbiota of rhesus monkeys showed a gradual increase in Betaproteobacteria with age (young, young adult, adult, and old) at the class level (Adriansjach et al. 2020). The present MR study provides evidence of increased levels of Betaproteobacteria in the gut of frail older patients. A retrospective cohort study has shown that *C. innocuum* causes worse remission in patients with inflammatory bowel disease (IBD), which is associated with intestinal stenosis (Le et al. 2022). Furthermore, 39%, 49%, and 12% of the patients with IBD were at no‐, low‐, and high risk of frailty, respectively, suggesting that older patients with IBD are more likely to be frail (Kochar et al. 2022). This cohort study showed that frailty is positively associated with the risk of hospitalization and death among older patients with IBD. Notably, it directly identified *C. innocuum*‐induced exacerbation of frailty. This highlights the need for clinicians to monitor for *C. innocuum* infections in hospitalized frail patients with IBD and consider probiotic interventions to mitigate frailty and prevent hospitalizations.

We also observed differences in gut permeability and fecal microbiota between frail and healthy older adults. Frail individuals had higher levels of *Bifidobacterium* and *Akkermansia*, lower levels of *Prevotella*, alongside increased inflammatory cytokines like IL‐6 and C‐reactive protein (Xu et al. 2021). *Prevotella*, a prevalent microorganism in healthy guts, has anti‐inflammatory properties and produces SCFAs beneficial for gut health (Almeida et al. 2022; Tett et al. 2019; Ley 2016; Laursen et al. 2017; Louis and Flint 2017). These findings suggest that variations in *Bifidobacterium*, Bacteroidetes, and related microbial profiles play crucial roles in frailty, with possible genetic links enhancing our understanding of these relationships (Kong et al. 2016; Biagi et al. 2016). In addition, changes in *Allisonella*, which have been associated with type 2 diabetes, further indicate a broader pattern of gut dysbiosis that could affect inflammation and frailty, though the precise mechanisms require further investigation (Neri‐Rosario et al. 2023).

In terms of dietary influences, our study found that a long‐term Mediterranean diet enhanced the abundance of *Eubacterium* strains (*E. ectale*, *E. eligens*, and *E. xylanophilum*), reducing frailty and boosting cognitive function in older adults (Ghosh et al. 2020). Although *Eubacterium* is generally linked with good health and is less abundant in frail individuals (Zhang et al. 2020), contrasting findings from a recent MR study suggest *E. coprostanoligenes* may increase hypertension risk, which correlates strongly with frailty (Y. Li, Fu, et al. 2023; Aprahamian et al. 2018). This study also indicated that certain *Eubacterium* strains could exacerbate frailty, highlighting discrepancies that may arise from complex gut flora interactions and the impact of factors like diet and medication on microbial composition. Thus, robust prospective studies are needed to clarify these relationships. In addition, a model by Aranaz et al. associated higher *Allisonella* levels with increased inflammation (Aranaz et al. 2021), yet this study found no clear causal impact of frailty on gut microbiota, suggesting possible reverse causation or confounding effects from earlier observations.

We also identified *TET2*, as a hub gene that links frailty and microbial profiles. Mice with disrupted *TET2* at the thymocyte developmental stage exhibit a distinct disease phenotype, resulting in impaired development, migration, and function of several T cell subsets, such as the aberrant proliferation of iNKT cells, the inability of CD8+ cells to migrate to the periphery, and dysfunctional regulatory T cells (Tregs) (Tsagaratou et al. 2017). Similarly, Nakatsukasa et al. found that *TET2* is important for Tregs stability and immune homeostasis. Notably, *TET2* deficiency in aged Tregs significantly elevated serum levels of immunoglobulins (Ig) IgG1, IgG3, IgM, and IgE and IL‐17‐producing cells (Nakatsukasa et al. 2019). Recent studies have shown that *TET2* mutations associated with aging lead to bone marrow expansion and dysregulation of innate immunity, promoting cancer and cardiovascular disease development (Ferrone et al. 2020). In addition, whole‐genome sequencing of germ‐free mice revealed that the exposed gut microbiota was mediated by *TET2/3*, leading to changes in gut homeostasis and inflammation (Ansari et al. 2020). Consistent with these findings, our ssGSEA results showed that frail individuals exhibited lower antimicrobial resistance, mediated by innate immune dysfunction. Spearman correlation analysis indicated a significant relationship between gut bacteria and *TET2* expression in frail patients. Therefore, MR showed that the potential mechanism of causality between the flora and frailty may involve *TET2*‐mediated innate immune modulation (Gao et al. 2025).

This MR study elucidated the bidirectional causal relationship between gut flora and frailty, offering several advantages: it reduced bias from confounders and reverse causality, utilized a large GWAS‐pooled dataset with SNPs closely linked to the studied traits, and ensured no sample overlap between exposure and outcome groups, enhancing the accuracy of the estimates. Furthermore, the integration of genomic and transcriptomic data deepens our understanding of the interactions between phenotypic and functional genes, aiding further exploration of the gut microbiota's impact on frailty.

However, there are notable limitations to this study. The majority of participants were of European descent, limiting the applicability of the findings to other populations. The functions of the selected SNPs remain underexplored, potentially overlooking some pleiotropic effects. While the GWAS on intestinal flora was extensive, the sample size and scope of loci studied are still limited, necessitating further studies with larger sample sizes and comprehensive RNA expression data for validation. Finally, given that frailty predominantly affects older adults and gut microbiota composition varies with age, the MR estimates may not fully reflect the age‐specific clinical impacts of microbiota on frailty.

## 5 Conclusions

In conclusion, our study suggests that *TET2* may mediate the effect of the gut microbiota on frailty in older patients, likely through modulation of the innate immune system. These findings underscore the potential for gut microbiota manipulation—such as through dietary interventions, probiotics, or other microbiome‐targeted therapies— as a preventive and therapeutic strategy for frailty in older patients. Future research should aim to further explore the precise mechanisms by which *TET2* and the gut microbiota interact, as well as the clinical applications of these insights in managing frailty and improving the health of aging populations.

## Author Contributions

**Xinlei Hou**: writing – original draft, writing – review and editing, supervision, funding acquisition, validation. **Luwen Zhu**: conceptualization, supervision, writing – original draft, writing – review and editing, project administration. **Jiongliang Zhang**: investigation, methodology. **Xinyue Li**: methodology, data curation. **Donghui Yu**: data curation. **Yuting Wang**: data curation, formal analysis. **Yumeng Su**: visualization, methodology. **Xiangyu Wei**: investigation, visualization. **Hanwen Ma**: software, data curation. **Wenjing Song**: software, data curation. **Jinting Li**: data curation. **Lili Teng**: data curation, methodology. **Qiang Tang**: validation, conceptualization, writing – review and editing, resources. **Minmin Wu**: conceptualization, writing – original draft, writing – review and editing

## Ethics Statement

This investigation utilized publicly accessible GWAS summary data; the original GWAS had obtained informed consent and undergone ethical review.

## Conflicts of Interest

The authors declare no conflicts of interest.

## Peer Review

The peer review history for this article is available at https://publons.com/publon/10.1002/brb3.70657

## Supporting information



Additional file 1**: Table S1**. Included instrumental variables for significant taxa. **Table S2**. MR analysis results of all eight methods for significant taxa. **Table S3**. Included instrumental variables for frailty. **Table S4**. DEGs in frailty and robust. **Table S5**. Gene‐set enrichment analysis. **Table S6**. Top 10 genes in largest connected network ranked by MCC method.

## Data Availability

Publicly available datasets were analyzed in this study. The dataset(s) supporting the conclusions of this article are available in the MiBioGen [https://mibiogen.gcc.rug.nl/] and UK Biobank [https://www.ukbiobank.ac.uk/].
